# Iowa

**Published:** 1897-02

**Authors:** 


*Iowa.*—It is thought that the ravages of hog cholera in this State will entail a loss of 2,000,000 hogs, one-third of the number raised. Another extensive outbreak now prevailing extends over western Illinois and northeastern Missouri, carrying off thousands of hogs.

